# Correction: Ecotoxicological prediction of organic chemicals toward *Pseudokirchneriella subcapitata* by Monte Carlo approach

**DOI:** 10.1039/d2ra90123d

**Published:** 2022-12-01

**Authors:** Shahram Lotfi, Shahin Ahmadi, Parvin Kumar

**Affiliations:** Department of Chemistry, Payame Noor University (PNU) 19395-4697 Tehran Iran Sh.lotfi@pnu.ac.ir; Department of Pharmaceutical Chemistry, Faculty of Pharmaceutical Chemistry, Tehran Medical Sciences, Islamic Azad University Tehran Iran ahmadi.chemometrics@gmail.com; Department of Chemistry, Kurukshetra University Kurukshetra Haryana 136119 India

## Abstract

Correction for ‘Ecotoxicological prediction of organic chemicals toward *Pseudokirchneriella subcapitata* by Monte Carlo approach’ by Shahram Lotfi *et al.*, *RSC Adv.*, 2022, **12**, 24988–24997, https://doi.org/10.1039/D2RA03936B.

The authors regret the omission of one of the co-corresponding authors’ email addresses from the original manuscript. The corrected email addresses are shown below.

The Royal Society of Chemistry apologises for these errors and any consequent inconvenience to authors and readers.

## Supplementary Material

